# Synthesis of Selected Mixed Oxide Materials with Tailored Photocatalytic Activity in the Degradation of Tetracycline

**DOI:** 10.3390/ma14185361

**Published:** 2021-09-17

**Authors:** Katarzyna Siwińska-Ciesielczyk, Angelika Andrzejczak, Dominik Paukszta, Adam Piasecki, Dariusz Moszyński, Agnieszka Zgoła-Grześkowiak, Teofil Jesionowski

**Affiliations:** 1 Institute of Chemical Technology and Engineering, Faculty of Chemical Technology, Poznan University of Technology, Berdychowo 4, PL-60965 Poznan, Poland; andrzejczak194@wp.pl (A.A.); dominik.paukszta@put.poznan.pl (D.P.); teofil.jesionowski@put.poznan.pl (T.J.); 2 Institute of Materials Science and Engineering, Faculty of Mechanical Engineering and Management, Poznan University of Technology, Jana Pawla II 24, PL-60965 Poznan, Poland; adam.piasecki@put.poznan.pl; 3 Institute of Inorganic Chemical Technology and Environment Engineering, Faculty of Chemical Technology and Engineering, West Pomeranian University of Technology in Szczecin, Piastów 42, PL-71065 Szczecin, Poland; dmoszynski@zut.edu.pl; 4 Institute of Chemistry and Electrochemistry, Faculty of Chemical Technology, Poznan University of Technology, Berdychowo 4, PL-60965 Poznan, Poland; agnieszka.zgola-grzeskowiak@put.poznan.pl

**Keywords:** TiO_2_, ZrO_2_, ZnO, mixed oxide materials, photodegradation, tetracycline

## Abstract

The elimination of antibiotics occurring in the natural environment has become a great challenge in recent years. Among other techniques, the photocatalytic degradation of this type of pollutant seems to be a promising approach. Thus, the search for new photoactive materials is currently of great importance. The present study concerns the sol–gel synthesis of mono, binary and ternary TiO_2_-based materials, which are used as active photocatalysts. The main goal was to evaluate how the addition of selected components—zirconium dioxide (ZrO_2_) and/or zinc oxide (ZnO)—during the synthesis of TiO_2_-based materials and the temperature of thermal treatment affect the materials’ physicochemical and photocatalytic properties. The fabricated mixed oxide materials underwent detailed physicochemical analysis, utilizing scanning-electron microscopy (SEM), X-ray diffraction (XRD), diffuse reflectance spectroscopy (DRS), energy-dispersive X-ray spectroscopy (EDS), low-temperature N_2_ sorption (BET model), X-ray photoelectron spectroscopy (XPS) and Fourier transform infrared spectroscopy (FTIR). The synthesized mixed oxide materials were used as photocatalysts in the heterogeneous photodegradation of tetracycline (TC). The physicochemical properties of the fabricated photocatalysts, including morphology, crystalline and textural structure, as well as the pH of the reaction system in the photocatalytic tests, were taken into account in determining their photo-oxidation activity. LC–MS/MS analysis was used to identify the possible degradation products of the selected antibiotic.

## 1. Introduction

The dynamic development of civilization and technological progress contribute to the growing problem of increasing quantities of pollutants generated into the natural environment. Among a broad range of such substances, residues of medical and pharmaceutical preparations and products have been recognized as a significant global problem [1]. Among the wide variety of pharmaceuticals and personal care products (PPCPs), tetracyclines are the most produced and consumed [2]. Therefore, one of the challenges currently being taken up in many scientific centers around the world is the development of new, effective methods for eliminating or removing various types of pollutants [3]. According to the available literature, methods such as adsorption [4,5], membrane techniques [6], electrocoagulation [7], electrochemical methods [8] and advanced oxidation processes [9,10,11] have been applied for the elimination of certain kinds of antibiotics, include tetracyclines. Very often, for the removal of antibiotics in wastewater, adsorption [4,5] or membrane techniques [6] have been applied; however, it should be noted that these methods do not break down the impurities, but only transfer them between phases. In recent years, many researchers have proposed photocatalysis [9,10,11] as an alternative strategy for the elimination of tetracyclines, because of the high efficiency of purification that it offers, as well as the fact that PPCPs are very sensitive to light irradiation. Moreover, heterogeneous photocatalysis, which is classed among the advanced oxidation processes, enables the mineralization and transformation of pollutants into compounds exhibiting greater biodegradability, finally leading to CO_2_ and H_2_O [12].

In heterogeneous photocatalysis, the choice of a material which will be active and effective in the photocatalytic degradation of a given type of impurity is of key importance. In studies relating to the photocatalytic elimination of pollutants, materials containing TiO_2_ in their structure are frequently considered [13]. Many scientific reports identify TiO_2_ as the most satisfactory photoactive material, and it is applied in the photocatalytic elimination of various types of impurities present in the natural environment. However, it should be noted that this material is also characterized by fast recombination of photo-excited electron–hole pairs, a wide energy gap, a low quantum yield of the process and the ability to absorb only ultraviolet radiation. These features limit its practical application in photocatalytic processes [14,15]. To eliminate these disadvantages, many research centers worldwide are focused on the development of effective methods of producing TiO_2_-based materials with unique physicochemical properties, including defined phase composition, crystallite size or BET surface area, which are factors in their high and constantly growing popularity in photocatalytic processes [16]. Unquestionably, the synthesis of semiconductor heterostructure systems produces new materials having quite different properties than the original components, especially new crystallographic phases. Moreover, the fabrication of this type of product changes its surface properties, which depend on the characteristics of the selected components. New sites are formed in the interface between selected oxides, which are responsible for changing the energy levels in the band gap region, as well as creating a new photoactive crystalline phase. Moreover, the combination of two semiconductor materials provides an opportunity to enhance charge separation, suppress charge recombination and broaden the spectral range of light absorption [17,18].

Among different methods described in the literature for improving the photo-oxidation activity of photocatalysts, the synthesis of semiconductor/semiconductor heterostructures has been proposed. This solution strengthens the separation of photogenerated electrons (e^–^) and holes (h^+^) and also decreases their recombination rate [17,18]. One of the components which exhibit similar physicochemical properties to TiO_2_, and which can be combined with it, is ZnO. This compound offers features such as thermal and chemical stability, biocompatibility, non-toxicity, low cost and ease of synthesis. Moreover, ZnO absorbs a greater fraction of solar radiation than TiO_2_ [19]. The previous literature data indicate that the synthesis of mixed oxide materials consisting of TiO_2_ and ZnO can enhance the optical properties of titania, for example by broadening the range of light absorption, enhancing the separation of charge carriers and decreasing the recombination rate of electrons and positively charged holes, which are crucial in the photo-oxidative activity of photocatalysts [20,21,22,23,24]. Moreover, in the available literature, many works can be found concerning the fabrication of TiO_2_-ZnO materials, in which commercial TiO_2_ and ZnO are used as source materials or one of the component is firstly synthesized, and then another is deposited onto it. For instance, Prasannalakshmi and Shanmugam [20] have proposed the methodology in which TiO_2_ was combined with zinc oxide during sol–gel method—firstly TiO_2_ was synthesized and furthermore it was poured into aqueous solution of zinc acetate. As prepared materials were used as active photocatalysts in the degradation of two selected organic dyes. Due to relatively high surface area, rod-like morphology, reduced band gap and low charge transfer resistance, prepared TiO_2_/ZnO materials exhibited enhanced photoactivity towards degradation of analyzed organic dyes. On the other hand, Das et al. [22] have demonstrated the innovative approach to fabricate ZnO-TiO_2_ heterojunction. Authors have applied commercial TiO_2_ (Degussa P25) and zinc(II) nitrate hexahydrate, which were combined via hydrothermal treatment in order to obtain active ZnO-TiO_2_ heterojunction, which exhibits improved photocatalytic properties in degradation of organic dyes. Moreover, García-Ramírez et al. [24] have reported preparation of TiO_2_-ZnO thin films by the sputtering technique on glass substrates. Mentioned method leads to the formation of ZnTiO_3_ and Zn_2_TiO_4_ phases, which play a crucial role in photocatalytic applications. ZrO_2_ is another semiconductor that can be coupled with TiO_2_ to enhance its physicochemical and functional properties. The main advantage of the addition of ZrO_2_ to TiO_2_ is that it increases the mobility of electrons and stabilizes the phase transformation of anatase into rutile, thus increasing the material’s thermal stability. Moreover, the forceful correlation between these two components leads to the creation of new active catalytic sites, which is important in the photocatalytic approach [25,26,27,28,29]. Pirzada et al. [25] synthesized a crystalline and homogeneous TiO_2_/ZrO_2_ photocatalyst nanocomposites via a hybrid sol–gel process. The fabricated composites have shown an efficient photocatalytic activity for degradation of the organic pollutants. Li et al. [26] reported the fabrication of ZrO_2_-TiO_2_ composites with macroporous structures and controlled ZrO_2_ content via sol–gel method. Obtained composites exhibited remarkably higher photocatalytic activities than the pure TiO_2_ and ZrO_2_. Pentelides [29] reported that doping with Zr leads to the creation of stable electron–hole pairs; it has the effect of trapping electrons and positive holes, thus suppressing their recombination. 

In the present work, mono, binary and ternary TiO_2_-based materials were synthesized. To the best of our knowledge, the novelty aspects of this work concern the methodology of obtaining of TiO_2_-based systems via sol–gel method combined with a heat-treating process, in which synthesis of mentioned components of oxide system was simultaneously performed in one reaction medium by using their organic precursors. The advantage of such methodology is effective combination of selected oxide components via chemical interaction during hydrolysis and their condensation. The main aim was to investigate how the addition of selected components during synthesis, and the temperature of thermal treatment, affect the morphology, crystalline and textural structure, chemical composition and band gap energy of the fabricated oxide systems. The synthesized materials were then used as active photocatalysts in the heterogeneous photodegradation of tetracycline. In determining the photo-oxidative activity of the fabricated photocatalysts, their physicochemical properties, such as morphology, crystalline and textural structure, as well as the pH of the reaction system in the photocatalytic tests, were taken into consideration. A possible mechanism of degradation of the selected antibiotic in the presence of the fabricated photocatalysts was also proposed.

## 2. Materials and Methods

### 2.1. Chemicals and Materials

Titanium(IV) isopropoxide (TTIP, 97%), zirconium(IV) propoxide (TZIP, solution 70 wt.% in 1-propanol) and tetracycline (TC, 98%) were acquired from Sigma-Aldrich (St. Louis, MO, USA). Zinc acetate dihydrate (ZA, 99.5%) was purchased from Merck KGaA (Darmstadt, Germany). Ammonia (25%) and propan-2-ol (IPA, 99.5%) were purchased from Chempur (Piekary Śląskie, Poland). Deionized water was used in all experiments. All reagents were used without any further purification.

### 2.2. Fabrication of Mixed Oxide Systems 

**Figure 1:**
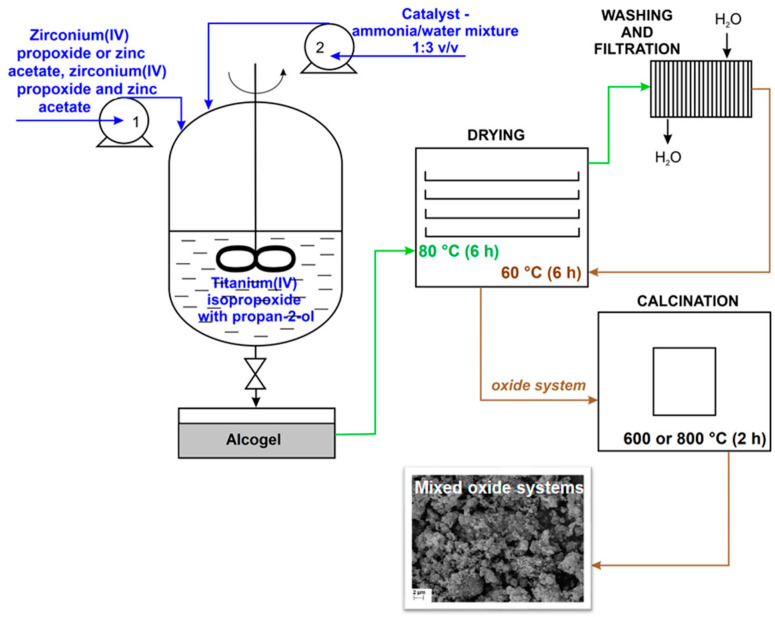
Scheme of sol–gel synthesis of mixed oxide photocatalysts.

The mono, binary and ternary oxide systems based on TiO_2_, ZrO_2_ and/or ZnO (with a TiO_2_:ZrO_2_ or TiO_2_:ZnO molar ratio of 8:2, or a TiO_2_:ZrO_2_:ZnO molar ratio of 8:1:1) were fabricated by the sol–gel route, according to the modified methodology reported in our previous work [30]. First, to an alcohol solution of TTIP, which was placed in a three-neck flask, the required quantities of ZrO_2_ and/or ZnO precursors were introduced. The resulting mixture was stirred (1000 rpm) at room temperature. In the next step, ammonia solution was added until the pH reached 8. In this way, “wet” gel is formed (labeled as alcogel). Finally, the resultant mixture (alcogel) was subjected to aging, drying, washing, filtration, drying and calcination. The scheme of synthesis of mixed oxide photocatalysts is presented in Figure 1. 

### 2.3. Characterization of Synthesized Photocatalysts 

The microstructure and surface morphology of the fabricated products were determined by scanning electron microscopy (SEM), using an EVO40 apparatus (Zeiss, Jena, Germany). To verify the crystallinity of the synthesized photocatalysts, X-ray powder diffraction analysis (XRD) was recorded by a TUR-M62 diffractometer (Carl Zeiss, Jena, Germany—Cu Kα radiation, α = 1.5418 Å, Ni filtered, Δ2θ = 0.04°, 2θ = 10–80°). To measure the optical properties of photocatalysts in the range of 200–800 nm, an Evolution 220 UV–Vis/DR spectrophotometer (Thermo Fisher Scientific Inc., Waltham, MA, USA), fitted with a photometric ball and using BaSO_4_ as a reference, was applied. An ASAP 2020 physisorption analyzer (Micromeritics Instrument Co., Norcross, CA, USA) was used to determine the textural properties of the fabricated photocatalysts. Prior to analysis, the synthesized photocatalysts were degassed at 120 °C for 4 h. The measurements were performed by using N_2_ at −196 °C. To verify the chemical composition of mixed oxide systems (contents of Ti, Zr, Zn and O), energy dispersive X-ray spectroscopy (EDS) was performed, using a Princeton Gamma-Tech unit equipped with a prism digital spectrometer (Princeton Gamma-Tech, Princeton, NJ, USA). The surface composition of photocatalysts was investigated by X-ray photoelectron spectroscopy (XPS) with Mg Kα (*h*ν = 1253.7 eV) radiation, using a Prevac (Rogów, Poland) system equipped with a Scienta SES 2002 electron energy analyzer operating at constant transmission energy (*Ep* = 50 eV). The samples were placed in a silver-plated titanium sample holder. The analysis chamber during experiments was evacuated to a vacuum of about 1 × 10^−9^ mbar. Charging effects were corrected by using the O 1s peak maximum set to 530.0 eV. The reproducibility of the peak position thus obtained was ±0.1 eV. Fourier transform infrared analysis (FTIR), using a Vertex 70 apparatus (Bruker, Karlsruhe, Germany), was performed to identify the presence of functional groups in the synthesized photocatalysts.

### 2.4. Photo-Oxidation Activity of Synthesized Photocatalysts

The photocatalytic elimination of tetracycline was performed in a UV-RS2 type laboratory reactor (Heraeus, Hanau, Germany) manufactured from borosilicate glass. The degradation of tetracycline took place at room temperature at pH values of 3, 6 and 9, using the mixed oxide materials as photocatalysts. To obtain an acidic or alkaline environment, HCl or NaOH solution was added to the tetracycline solution. A medium-pressure mercury lamp (150 W) was used as a UV light source. The heterogeneous photocatalysis tests were carried out as follows: At the first step, in 100 cm^3^ of tetracycline at an initial concentration of 10 mg/dm^3^, 20 mg of the photocatalyst was suspended. Before illumination, the prepared reaction mixture was magnetically stirred (R05 IKAMAG magnetic stirrer, IKA Werke GmbH, Staufen im Breisgau, Germany) in darkness for 30 min to reach the sorption equilibrium. After this time, the concentration of adsorbed tetracycline was measured. In the next step, to activate the photocatalytic process, the radiation was turned on. The illuminated reaction mixture was sampled from the reactor at regular intervals (5, 10, 15, 20, 25, 30, 35, 40, 60, 90 and 120 min) and centrifuged for 20 min to remove the photocatalyst. To analyze the absorbance of the sampled mixture at wavelengths of 356 nm (at pH 3 and 6) and 380 nm (at pH 9), a UV–Vis spectrophotometer (V-750, Jasco, Oklahoma City, OK, USA) was used. The concentration of adsorbed or degraded tetracycline was read off from the calibration curve: [Tetracycline] = 0.0331*Abs. The efficiency of elimination of the selected antibiotic (*W*(*%*)) was calculated according to the following formula:

(1) $$W(\%)=\left(1-\frac{C_{t}}{C_{0}}\right)\cdot 100\%$$

where *C*_0_ and *C_t_* are the concentrations of the antibiotic prior to and after irradiation, respectively.

### 2.5. Kinetic Study 

To determine the rate constants of the degradation process, as well as the half-life of tetracycline, a kinetic study was performed. The kinetics of photocatalytic decomposition of tetracycline were calculated according to the Langmuir–Hinshelwood equation:

(2) $$r=\frac{dC}{dt}=k\left(\frac{KC}{1+KC}\right)$$

Considering that the elimination of tetracycline is a pseudo-first-order reaction, the reaction rate constant can be determined as the slope of the linear regression:

(3) $$-\ln\left(\frac{C_{t}}{C_{0}}\right)=kt$$

where *k* is the rate of degradation of tetracycline (min^−1^), *K* is the equilibrium constant of adsorption of the antibiotic on the surface of the catalyst, and *C*_0_ and *C_t_* are the concentrations of tetracycline in aqueous solution before irradiation (*t* = 0) and after time, *t*.

Knowledge of the reaction rate constant, *k*, makes it possible to determine the half-life of the pharmaceutical pollutant (*t*_1/2_):

(4) $$t_{\frac{1}{2}}=\frac{\ln2}{k}$$

### 2.6. LC–MS/MS Determination of Degradation Products of Tetracycline

Identification of degradation products was performed by using an LC–MS/MS system consisting of an UltiMate 3000 UHPLC instrument from Dionex (Sunnyvale, CA, USA) and a 4000 QTRAP mass spectrometer from ABSciex (Foster City, CA, USA). For the analyses, a Gemini-NX C18 column (100 × 2.0 mm; 3 µm) from Phenomenex (Torrance, CA, USA) was used, thermostated at 35 °C. The injection volume was 5 µL, and the mobile phase consisted of 5 mM aqueous solution of ammonium acetate and acetonitrile at a flow rate of 0.3 cm^3^/min in the following gradient: 0 min = 50% acetonitrile, 2 min = 100% acetonitrile and 6 min = 100% acetonitrile. The effluent from the chromatographic column was directed to the mass spectrometer via an electrospray (ESI) ionization source operating in positive mode. Nitrogen was used in the electrospray source and the mass spectrometer. The operating parameters of the source and mass spectrometer were as follows: curtain gas 10 psi, nebulization gas 45 psi, auxiliary gas 45 psi, ESI temperature 450 °C, ESI voltage 4500 V and declustering potential 45 V. Chromatograms were collected in enhanced mass spectra mode in a 50–500 *m/z* range, enabling the fragmentation of selected ions.

## 3. Results and Discussion

### 3.1. Physicochemical Characterization of Photocatalysts

The first task in the physicochemical characterization of the photocatalysts concerned evaluation of the effect of the addition of selected components (ZrO_2_ and/or ZnO) and the temperature of thermal treatment on the morphology and microstructure of the fabricated mixed oxide materials. SEM images for all synthesized products (presented in the Supplementary Material (Supplementary Materials Figure S1)), showed similar morphology. Moreover, particles spherical and ovoid in shape, with diameters of 300–500 nm, exhibited a tendency to aggregate into irregular particles. This effect is visible in all of the synthesized products, and may be associated with the caking of the material [30,31,32]. Analysis of the SEM images indicated that neither the addition of the selected components nor their thermal treatment had a significant influence on the morphology of the fabricated products.

**Figure 2:**
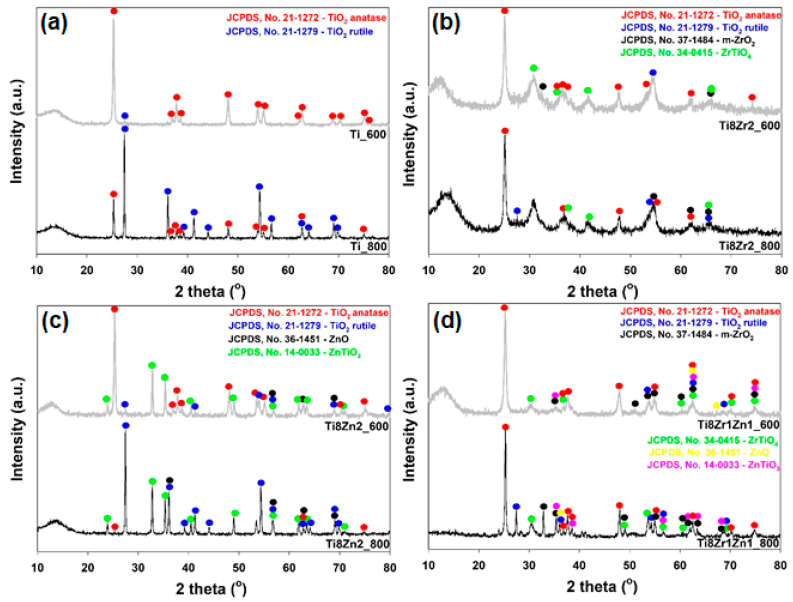
Crystalline structure of (**a**) Ti_600 and Ti_800, (**b**) Ti8Zr2_600 and Ti8Zr2_800, (**c**) Ti8Zn2_600 and Ti8Zn2_800, (**d**) Ti8Zr1Zn1_600 and Ti8Zr1Zn1_800 oxide photocatalysts.

In the next step, it was determined how the addition of the selected components to TiO_2_ and the temperature of thermal treatment affected the crystalline structure of the fabricated systems. The X-ray pattern for the Ti_600 sample (Figure 2a—grey curve) indicates the presence of TiO_2_ with an anatase structure (JCPDS no. 21-1272), which is evidenced by the existence of diffraction peaks at 2θ = 25.28, 36.95, 37.80, 38.58, 48.05, 53.89, 55.06, 62.12, 62.69, 68.76, 70.31, 75.03 and 76.02. For this sample there is also visible a diffraction peak at 2θ = 27.45 (with low intensity), which is specific to rutile. The diffractogram for the Ti_800 sample (Figure 2a—black curve) shows diffraction peaks characteristic for both anatase and rutile. The peaks at 2θ = 27.45, 36.09, 39.19, 41.23, 44.05, 54.32, 56.64, 62.74, 64.04, 69.01 and 69.79 are characteristic for the rutile structure (JCPDS no. 21-1279). For this sample, a decrease in the intensity of diffraction peaks specific to anatase was observed. Analysis of the obtained X-ray patterns shows that with increasing temperature of calcination, the less thermodynamically stable form of TiO_2_, i.e., anatase, is transformed into the most stable structure, i.e., rutile.

The diffractograms for the TiO_2_-ZrO_2_ oxide systems subjected to thermal treatment at 600 and 800 °C, respectively (samples Ti8Zr2_600 and Ti8Zr2_800—Figure 2b) show not only diffraction maxima originating from TiO_2_ (anatase and rutile), but also peaks related to the presence of the monoclinic structure of ZrO_2_ (m-ZrO_2_—JCPDS no. 37-1484). The X-ray patterns for the Ti8Zr2_600 and Ti8Zr2_800 samples also exhibited diffraction peaks at 2θ = 30.44, 32.61, 41.91 and 65.99, which can be assigned to the zirconium titanate (ZrTiO_4_) structure (JCPDS no. 34-0415). The presence of the ZrTiO_4_ crystalline structure indicates that the Ti atoms in the crystalline lattice have been partially replaced by Zr atoms. This fact confirms the obtainment of a mixed oxide system, and it also indicates that the formation of the Zr-O-Ti bond is enabled, which could, in turn, facilitate easy electron transfer across the oxide interfaces and enhance photocatalytic activity [33]. Moreover, the diffractogram for the Ti8Zr2_800 sample (the TiO_2_-ZrO_2_ oxide system synthesized at the molar ratio TiO_2_:ZrO_2_ = 8:2 and heat-treated at 800 °C) contains intense diffraction maxima indicating the presence of anatase, which confirms that the addition of ZrO_2_ organic precursor during the synthesis of TiO_2_-based materials shifts the temperature of the phase conversion of anatase to rutile. Lukáč et al. [34] also proved that the doping of TiO_2_ with ZrO_2_ shifted the anatase-to-rutile phase transformation to a higher temperature. This indicates that the anatase phase was stabilized due to the presence of Zr atoms in the TiO_2_ lattice, which prevent the formation and crystal growth of the rutile phase [35]. Yang and Ferreira [36] also reported that the incorporation of Zr^4+^ ions retarded the anatase-to-rutile phase conversion. This fact was also confirmed by Hanaor et al. [37].

The diffractogram for the TiO_2_-ZnO oxide system heat-treated at 600 °C (Figure 2c) contains intense diffraction peaks characteristic for the anatase crystalline structure, as well as individual diffraction maxima of lower intensity, derived from rutile. Additionally, for this material, the peaks at 2θ = 56.60, 62.86 and 67.96 confirm the presence of the ZnO-wurtzite crystalline structure (JCPDS no. 36-1451). Similar results were obtained by Pei and Leung [38], who synthesized two-component systems based on TiO_2_ and ZnO. The sample also gave diffraction peaks at 2θ = 23.86, 32.73, 35.25, 40.43, 48.92, 56.79, 61.76, 63.40 and 70.91, assigned to the zinc titanate (ZnTiO_3_) structure (JCPDS no. 14-0033) [39]. García-Ramírez et al. [24] reported that the presence of titanates has a significant influence on the photocatalytic properties of synthesized materials. The diffractogram for TiO_2_-ZnO oxide material heat-treated at 800 °C contains diffraction peaks characteristic for TiO_2_ (especially for rutile), wurtzite and ZnTiO_3_. The XRD pattern for the Ti8Zn2_800 sample, in contrast to the Ti8Zn2_600 sample, contains diffraction peaks characteristic for rutile. The X-ray patterns for the Ti8Zn2_600 and Ti8Zn2_800 samples show that the incorporation of ZnO species during the synthesis of mixed oxide systems based on TiO_2_ shifts the temperature of phase transformation of anatase to rutile towards lower temperatures. Comparing the XRD patterns for the Ti_800 and Ti8Zn2_800 samples, we observe that the Ti8Zn2_800 sample produces lower intensity diffraction peaks assignable to the anatase structure.

The XRD patterns for the Ti8Zr1Zn1_600 and Ti8Zr1Zn1_800 samples (Figure 2d) exhibit diffraction peaks, which can be assigned to the dominant phase—anatase—in both samples. The diffractogram for the TiO_2_-ZrO_2_-ZnO oxide system heat-treated at 800 °C (compared with the sample calcined at 600 °C) shows that the intensity of diffraction peaks characteristic for rutile increases with increasing temperature of calcination. The diffractograms obtained for the TiO_2_-ZrO_2_-ZnO oxide materials heat-treated at 600 and 800 °C respectively (samples Ti8Zr1Zn1_600 and Ti8Zr1Zn1_800) confirm the presence of the characteristic structures of ZrTiO_4_ (2θ = 30.44, 37.31, 60.79, 63.3, 70.51 and 75.44) and ZnTiO_3_ (2θ = 35.25, 63.40 and 74.68). Additionally, for the TiO_2_-ZrO_2_-ZnO system heat-treated at 800 °C, a better-developed crystalline structure was observed, as evidenced by the increased intensity of individual diffraction peaks. Moreover, it was found that the addition of ZrO_2_ and ZnO organic precursors during the synthesis of three-component oxide systems, and their calcination lead to a reduction in the temperature of transformation of anatase to the rutile crystalline structure. The results indicate that probably the addition of ZrO_2_ organic precursor inhibits the phase transformation of TiO_2_. Hussein et al. [40] also noted that the application of substitutional ions of transition metals seems to inhibit anatase-to-rutile phase transformation even at elevated temperature. Okada et al. [41] noted that when TiO_2_ is doped with cations with valence lower than 4+, acceleration of its phase transformation is observed. This occurs because the presence of these ions provides a charge compensation mechanism by the formation of vacancies that enhance the transport of atoms in the anatase structure. The opposite situation is observed when cations with valence higher than 4+ are used in the doping process: their presence retards the transformation by forming interstitial Ti^3+^ cations that suppress atomic transport in the anatase structure.

**Figure 3:**
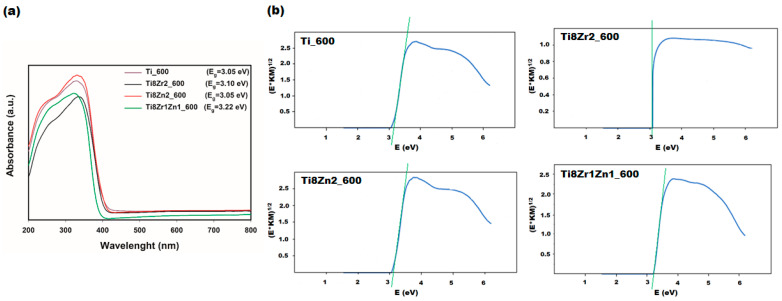
UV–Vis/DRS spectra (**a**) and Kubelka−Munk function (E*KM)^1/2^ as a function of photon energy (**b**) for Ti_600, Ti8Zr2_600, Ti8Zn2_600 and Ti8Zr1Zn1_600 materials.

The UV–Vis absorption spectra of Ti_600, Ti8Zr2_600, Ti8Zn2_600 and Ti8Zr1Zn1_600 samples were analyzed to understand the optical properties of selected fabricated photocatalysts (Figure 3). For the analyzed materials, absorption was observed in the range of UV radiation (250–380 nm) and in the visible light range (390–400 nm). Moreover, based on the Kubelka–Munk theory [42,43], the band gap energy of the photocatalysts was calculated. The band gap energies of the Ti_600, Ti8Zr2_600, Ti8Zn2_600 and Ti8Zr1Zn1_800 samples were 3.05, 3.10, 3.05 and 3.22 eV, respectively. The narrowing of the band gap from 3.2 eV for pure anatase to 3.05 eV for the Ti_600 sample is probably related to the presence of a small fraction of rutile (band gap 3.0 eV); this was confirmed by XRD analysis. The Ti8Zn2_600 sample has the same band gap energy as the pure TiO_2_ sample. The fact can be indicated that mentioned samples exhibited similar optical properties. In the case of Ti8Zr2_600 and Ti8Zr1Zn1_800 samples, the band gap was slightly blue shifted to 3.10 and 3.22 eV, respectively. The fact could be related to the formation of Zr-O-Ti bonds in mixed oxide systems, resulting from the substitution of Ti^4+^ with Zr^4+^ ions [33].

**Figure 4:**
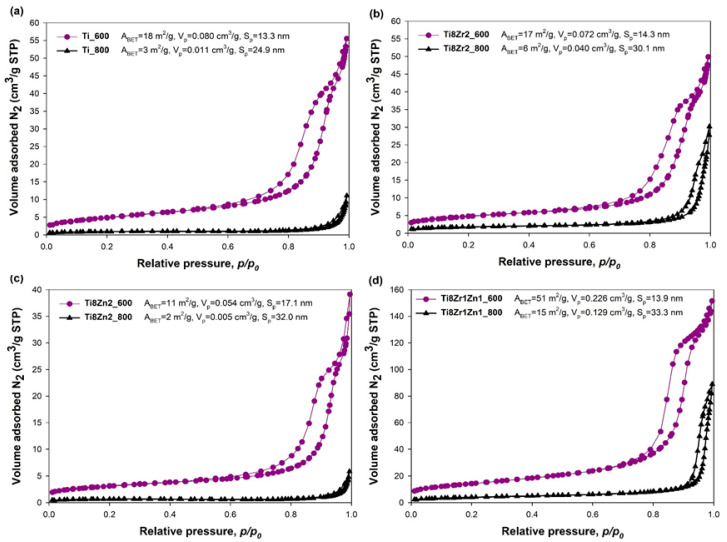
N_2_ adsorption/desorption isotherms of (**a**) Ti_600 and Ti_800, (**b**) Ti8Zr2_600 and Ti8Zr2_800, (**c**) Ti8Zn2_600 and Ti8Zn2_800, (**d**) Ti8Zr1Zn1_600 and Ti8Zr1Zn1_800 oxide photocatalysts.

To evaluate the textural properties, in particular the surface area (A_BET_), total pore volume (V_p_) and average pore diameter (S_p_), of the fabricated photocatalysts, low-temperature N_2_ sorption analysis was conducted. The N_2_ sorption isotherms of all synthesized oxide systems (see Figure 4) can be classified as type IV with type H3 hysteresis loop, corresponding to mesoporous materials—except for the Ti_800 and Ti8Zn2_800 samples. According to the IUPAC classification [44] the N_2_ sorption isotherms for the Ti_800 and Ti8Zn2_800 samples represented type II with type H4 hysteresis loop, typical for macroporous structures. 

The low-temperature N_2_ sorption results indicated that all photocatalysts heat-treated at 800 °C had lower BET surface area than those calcined at 600 °C. The decrease in surface area for samples heat-treated at 800 °C may be due to the phase composition of the fabricated systems, as well as greater caking of the grains and a tendency to form agglomerate structures [30,45,46]. Moreover, the pore size distributions for the fabricated photocatalysts shift toward larger structures (mesopores) in case of calcination at a higher temperature, which is related to the growth of crystallites and collapse of the porous structure parameters. The total pore volume followed the opposite trend to the BET surface area and the average pore diameter. 

The Ti8Zr1Zn1_600 sample had the highest value of A_BET_ (51 m^2^/g) among all of the fabricated photocatalysts. For this sample the value of S_p_ was 13.9 nm and V_p_ was 0.226 cm^3^/g. Binary oxide photocatalysts containing TiO_2_ and ZnO, heat-treated at 600 and 800 °C (samples Ti8Zn2_600 and Ti8Zn2_800—Figure 4c) exhibited the lowest BET surface area. For these photocatalysts, A_BET_ was equal to 11 and 2 m^2^/g for the materials heat-treated at 600 and 800 °C, respectively. The S_p_ values for these materials were 17.1 nm (sample Ti8Zn2_600) and 32 nm (sample Ti8Zn2_800), and the respective V_p_ values were 0.054 and 0.005 cm^3^/g. Konyar et al. [47] also noted that binary oxide systems synthesized with TiO_2_ and ZnO had several times smaller BET surface area than a pure TiO_2_ sample. A similar phenomenon was observed by Prasannalakshmi and Shanmugam [20]. Zhang et al. [48] also reported that the porous structure parameters of ZnO/TiO_2_ systems decreased with an increase in the crystallization temperature. However, in the case of Ti8Zr1Zn1_600 and Ti8Zr1Zn1_800 samples (ternary oxide systems), the BET surface area was higher by factors of approximately 2.8 and 5, respectively, than that of pure TiO_2_. Zheng et al. [49] also observed that the combination of ZnO with ZrO_2_ results in materials with higher BET surface area. Han et al. [50] similarly observed that TiO_2_ photocatalysts supported with ZnO and ZrO_2_ have higher BET surface area values than those containing only ZnO. They argued that Zr may hinder the sintering of pristine TiO_2_ crystallites upon high-temperature calcination. 

Chemical composition tests aimed at determining the mass fraction of individual elements in the fabricated oxide materials were performed applying the EDS method (see results presented in the Supplementary Materials Figure S2). The EDS results show that the conditions of heat treatment have no significant influence on the composition of the mixed oxide systems. In the case of binary and ternary oxide products containing ZrO_2_, independently of the temperature of calcination, the content of ZrO_2_ was two or three times higher than the assumed value, which confirms that the precursor of ZrO_2_ is hydrolyzed faster than the precursors of ZnO or TiO_2_. A similar observation was made by Aghabeyga and Khademi-Shamami [51]. The EDS results for the TiO_2_-ZrO_2_ materials confirmed that the samples contained TiO_2_ and ZrO_2_ (58.7% and 41.3% respectively in sample Ti8Zr2_600, and 59.3% and 40.7% in sample Ti8Zr2_800). The chemical composition results for the ternary oxide materials showed that sample Ti8Zr1Zn1_600 was composed of 60.9% TiO_2_, 29.8% ZrO_2_ and 9.3% ZnO, and sample Ti8Zr1Zn1_800 contained 61.5% TiO_2_, 29.9% ZrO_2_ and 8.6% ZnO. The EDS analysis for the TiO_2_-ZnO products confirmed that the contents of TiO_2_ and ZnO in the obtained materials were in accordance with the assumed molar ratio of the reactants.

Selected samples of TiO_2_, TiO_2_-ZrO_2_, TiO_2_-ZnO and TiO_2_-ZrO_2_-ZnO were analyzed by X-ray photoelectron spectroscopy. Since the O 1s spectra were used as a reference for binding energy correction, their maximum is located at 530.0 eV. However, the O 1s peak envelopes are virtually identical for all analyzed samples and differ only slightly in their width. The binding energy at 530.0 eV is characteristic for bulk oxygen ions observed for all reactant oxides: TiO_2_, ZrO_2_ and ZnO. Moreover, the ZnO 2p_3/2_ spectra observed for TiO_2_-ZnO and TiO_2_-ZrO_2_-ZnO samples are identical, with a maximum at a binding energy of 1021.6 eV. This energy position is reported for Zn^2+^ ions [52]. The maximum of the Zr 3d_5/2_ peak observed for both TiO_2_-ZrO_2_ and TiO_2_-ZrO_2_-ZnO samples is located at 182.2 eV. This position has been reported for Zr^4+^ ions in different compounds [30]. The Zr 3d_5/2_ lines differ only in peak width: the full width at half maximum (FWHM) of the peak observed for the TiO_2_-ZrO_2_ sample is 1.5 eV, while the value measured for the TiO_2_-ZrO_2_-ZnO sample is 1.9 eV.

**Figure 5:**
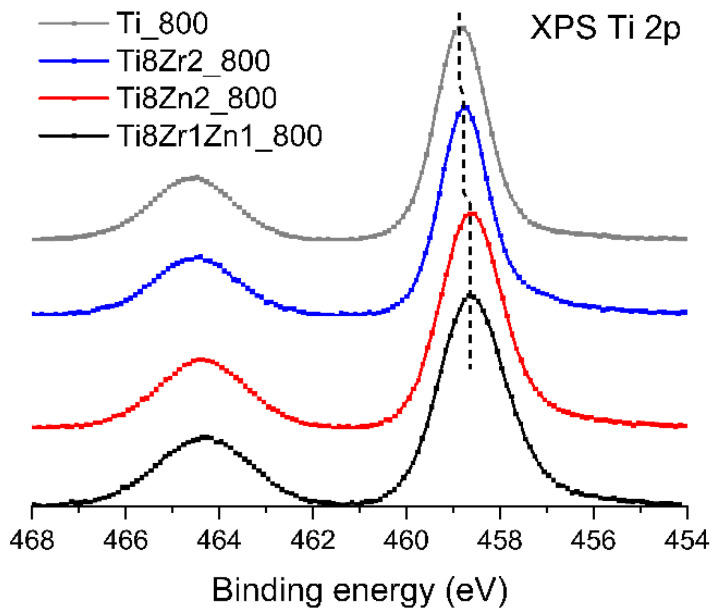
X-ray photoelectron spectra of Ti 2p region for selected samples. Intensities of photoelectron signals were normalized and overlayed with a vertical shift.

Minor, but systematic changes in the position of peak maxima are observed only in the XPS Ti 2p spectra, which are shown in Figure 5. The position of the Ti 2p_3/2_ component observed for the pure TiO_2_ sample is located at a binding energy of 458.8 eV, which is in agreement with the data reported for this material [53]. The maximum binding energy of the Ti 2p_3/2_ line observed for the TiO_2_-ZrO_2_ sample is shifted by 0.1 eV towards lower binding energy. Ti 2p_3/2_ lines recorded for both samples containing ZnO have the maximum shifted by 0.2 eV relative to the spectrum for pure TiO_2_. There is also a slight difference in the FWHM of the Ti 2p_3/2_ lines: the lines observed for TiO_2_ and TiO_2_-ZrO_2_ have a width of 1.4 eV, while the FWHM of lines observed for both samples containing ZnO is 1.7 eV. 

The observed variations in the positions and widths of XPS lines acquired for the analyzed samples are slight and may be a result of experimental error. However, it should also be noted that the shift of Ti 2p lines observed in the case of both samples with added ZnO may be a result of a defective TiO_2_ structure. When the TiO_2_ crystalline structure contains ions of a metal of lower valence than the Ti^4+^ ions, a shift of the Ti 2p spectrum towards lower binding energy is expected. This is what was observed in the present study. It is possible that Zn^2+^ ions are substituted for some of the Ti^4+^ ions in the TiO_2_ structure, and this is reflected in the XPS spectrum. In the case of ZrO_2_, the Zr^4+^ ions have the same valence as the Ti^4+^ ions, and this effect is not observed.

Fourier transform infrared spectroscopy (FTIR) was performed to confirm the effectiveness of the synthesis of mixed oxide systems based on the proposed methodology (Supplementary Materials Figure S3). On the FTIR spectra of the fabricated photocatalysts, stretching and bending vibrations of O–H bonds, characteristic for either water molecules or surface hydroxyl groups, were visible at wavenumbers 3400 and 1630 cm^−1^, respectively [30]. Moreover, for all fabricated photocatalysts there was a visible band at 2920 cm^−1^ attributed to C–H stretching vibrations, which originate from the alkoxide precursor and the solvent (propan-2-ol) applied during the synthesis [30]. Furthermore, for all analyzed materials, two strong bands were observed at 714 and 640 cm^−1^, which can be attributed to symmetric stretching vibrations of Ti–O–Ti groups [30,54]. In the case of binary and ternary oxide systems containing ZrO_2_, the FTIR spectra (Supplementary Materials Figure S3b,d) feature an absorption band at wavenumber 742 cm^−1^ indicating Zr–O groups [30], as well as a band at 721 cm^−1^ corresponding to the vibrations of Ti–O–Zr groups [30,55,56]. The FTIR spectra for TiO_2_-ZnO and TiO_2_-ZrO_2_-ZnO samples (Supplementary Materials Figure S3c,d) show an absorption peak typical for vibrations of Zn–O groups at 550 cm^−1^ [47]. There is also a visible band at wavenumber 750 cm^−1^, indicating the presence of Zn–O–Ti groups [47]. The presence of the bands at wavenumber 750 cm^−1^ (from vibrations of Ti–O–Zn groups) and 721 cm^−1^ (from vibrations of Ti–O–Zr groups) confirmed the effectiveness of the proposed methodology in the synthesis of mixed oxide systems.

### 3.2. Photocatalytic Degradation of Tetracycline

**Figure 6:**
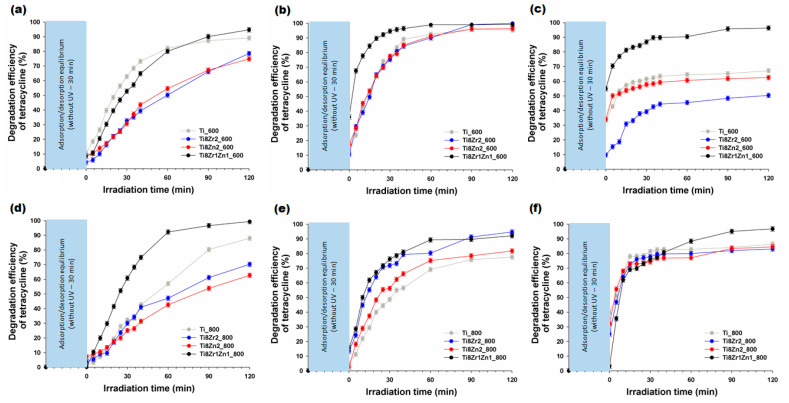
Photocatalytic elimination of TC in the presence of the synthesized photocatalysts at (**a**,**d**) pH 3, (**b**,**e**) pH 6 and (**c**,**f**) pH 9.

It is well-known that one of the crucial factors influencing the efficiency of a photocatalytic process is the pH. This is directly related to the surface charge of both the TC and the photocatalyst used. The main goal of the work presented here was to determine the photocatalytic activity of the synthesized photocatalysts in the degradation process of a model antibiotic –TC– under the influence of UV radiation. The degradation efficiencies under UV irradiation in the presence of the fabricated photocatalysts at different pH values are presented in Figure 6.

The results of photocatalytic tests showed that the decomposition of TC at pH 3 in the presence of photocatalysts calcined at 600 °C (Figure 6a) was fastest for the Ti8Zr1Zn1_600 and Ti_600 samples (the maximum degradation after 120 min was 94.7% and 89.1%, respectively, while in the presence of the Ti8Zr2_600 and Ti8Zn2_600 materials the efficiency of degradation of TC was 78.5% and 74.8%, respectively). The photocatalytic elimination of the selected antibiotic at pH 3 in the presence of the Ti8Zr1Zn1_800 sample (Figure 6d) indicated that this photocatalyst exhibited higher photo-oxidative activity (the maximum degradation after 120 min was 99.4%) than pure Ti_800 (for which the maximum degradation after 120 min was 87.9%). The binary oxide photocatalysts obtained by combining TiO_2_ with ZrO_2_ or ZnO exhibited lower photocatalytic activity in the decomposition of TC. The maximum degradation efficiency was 70.2% for sample Ti8Zr2_800 and 62.7% for sample Ti8Zn2_800.

In the next step, the photocatalytic activity of the fabricated photocatalysts in the elimination of TC was measured at pH 6 (Figure 6b,e). The results of these tests for the photocatalysts heat-treated at 600 °C (Figure 6b) showed that after 120 min of UV irradiation, the yields of antibiotic elimination in the presence of the fabricated photocatalysts were at the same level (the degradation efficiency in all cases was higher than 95.0%). The results for the photocatalysts heat-treated at 800 °C (Figure 6e) showed that the efficiency of TC elimination in the presence of Ti8Zr2_800 and Ti8Zr1Zn1_800 samples was 94.7% and 92.1% respectively after 120 min of irradiation. However, the Ti_800 and Ti8Zn_800 samples exhibited a lower efficiency of degradation of the selected antibiotic at pH 6. The yields of TC degradation in the presence of the Ti_800 and Ti8Zn_800 materials were 77.5% and 81.7%, respectively.

In the last stage, the photocatalytic degradation of TC was carried out at pH 9 (Figure 6c,f). The results of these photocatalytic tests for the photocatalysts heat-treated at 600 °C (Figure 6c) showed that the highest efficiency of TC decomposition was attained by the Ti8Zr1Zn1_600 sample (after 120 min of UV irradiation, 96.3% of the antibiotic was decomposed). Samples Ti_600, Ti8Zr2_600 and Ti8Zn2_600 exhibited lower photocatalytic activity in the decomposition of the selected antibiotic. The maximum efficiency of degradation of TC was 67.2% and 62.5% in the presence of the samples Ti_600 and Ti8Zn2_600, respectively. A slightly lower degradation efficiency (50.4%) was recorded in the case of photocatalysis, using sample Ti8Zr2_600. The tests of the photo-oxidative activity of the photocatalysts heat-treated at 800 °C in the decomposition of TC at pH 9 indicated that all of the materials exhibit good photocatalytic activity (Figure 6f). The maximum degradation efficiency was 96.8% in the presence of sample Ti8Zr1Zn1_800, and slightly lower (86.4%, 83.1% and 84.5%) in the case of photocatalysis using samples Ti_800, Ti8Zr2_800 and Ti8Zn2_800, respectively.

The results of the photocatalytic tests showed that the photocatalysts with higher BET surface area exhibited higher photocatalytic activity in the elimination of TC. It is well-known that a larger surface area means greater possible adsorption of pollutants on the surface of a photocatalyst [19]. Moreover, materials with higher BET surface area, which are used in photocatalytic process, will exhibit higher photocatalytic activity, because during this process photogenerated electrons together with oxygen from the surroundings can be transformed into highly reactive superoxide radical anions (O_2_^–•^) [19]. Carević et al. [19] noted that the addition of ZrO_2_ leads to form of OH^–^ groups which increase in surface acidity. During photocatalytic process, OH^–^ groups are transformed to OH^•^ radicals as a result of the reaction with photogenerated holes. On the other hand, the higher efficiency of fabricated materials in degradation of selected impurity is evidenced with the present of ZrTiO_4_ phase.

The photocatalytic tests proved that the photocatalytic degradation of TC can be realized in a broad pH range. However, photocatalytic elimination of the selected antibiotic in the presence of the fabricated photocatalysts was most effective at pH 6. This has also been reported in other studies [57]. Zhu et al. [58] noted that the surface properties of a photocatalyst and speciation of TC significantly depend on the pH of the solution. It is well-known that the interaction and affinity between a photocatalyst and TC molecules vary with the solution pH. At a low pH, such as 3, TiO_2_-based photocatalysts are positively charged. When the pH of the solution is below 3.3, TC exists as a cation, +00, due to protonation of the dimethyl ammonium group. In this situation, the electrostatic repulsion between the positively charged TiO_2_-based photocatalysts hinders the adsorption of TC on their structure, resulting in lower degradation efficiency [7,58]. At a pH between 3.3 and 7.7, the zwitterion form of TC, +−0, predominates due to the loss of a proton from the phenolic diketone moiety. This means that, in this pH range, TiO_2_-based photocatalysts are characterized by neutral surface hydroxyl groups [7]. Figueroa et al. [59] suggested that the surface complexes of zwitterionic species of TC in this pH range are the major contributor to the mechanisms of adsorption onto inorganic materials. Similar observations were made by Chen et al. [60] and by Gu and Karthikeyan [61]. At a pH greater than 7.7, TC prevails as a monovalent anion +−−, or as a divalent anion 0−−, from the loss of protons from the tricarbonyl system and phenolic diketone moiety [7]. The fact that the efficiency of removal of TC is lower at pH 9 than at pH 6 can be attributed to electrostatic repulsion between the negatively charged TiO_2_-based photocatalysts and anionic species of TC [7,58].

**Figure 7:**
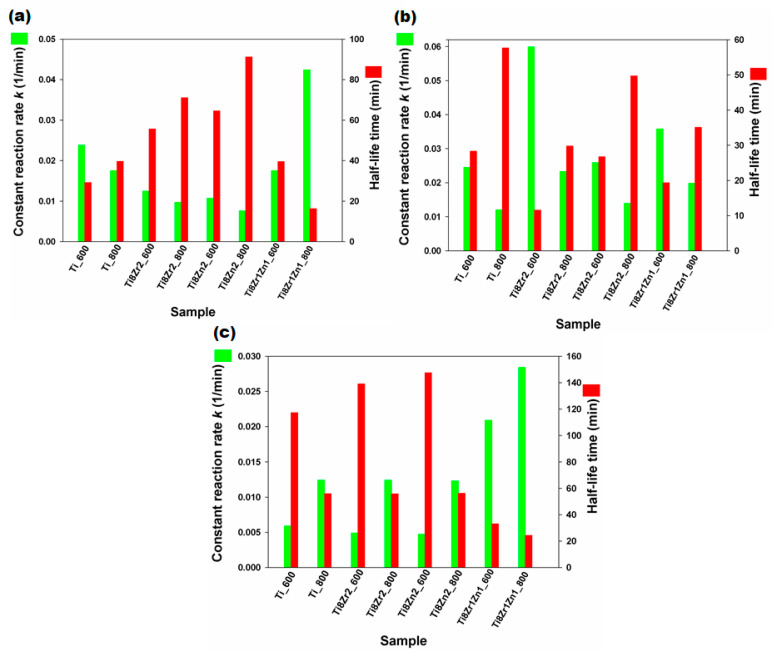
Values of *k* and *t*_1/2_ in the photocatalytic degradation of TC carried out at (**a**) pH 3, (**b**) pH 6 and (**c**) pH 9.

Kinetic studies of the photocatalytic elimination of the selected antibiotic show significant differences in the rate of degradation of the impurity with the use of the fabricated photocatalysts (Figure 7). In the photocatalytic tests carried out under acidic conditions (pH 3—Figure 7a), the highest value of *k* (0.0424 min^−1^) and the shortest *t*_1/2_ (16.034 min) were obtained for the Ti8Zr1Zn1_800 sample. The lowest value of *k* (0.0076 min^−1^) and the longest *t*_1/2_ (91.24 min) were recorded for the Ti8Zn2_800 sample.

In the photocatalytic degradation of TC carried out at pH 6 (Figure 7b) the highest value of *k* (0.0599 min^−1^) was obtained when the process was carried out in the presence of Ti8Zr2_600. The *t*_1/2_ value for this sample was 11.56 min. The lowest value of *k* (0.0120 min^−1^) was obtained when the degradation process was performed by using the Ti_800 sample, for which *t*_1/2_ was 57.67 min.

In the photocatalytic tests carried out at pH 9 (Figure 7c), the highest value of *k* (0.0284 min^−1^) and the shortest decomposition time *t*_1/2_ (24.38 min) were obtained for the Ti8Zr1Zn1_800 sample. The lowest value of *k* (0.0047 min^−1^) and the longest *t*_1/2_ (147.53 min) were recorded for the Ti8Zn2_600 sample.

### 3.3. Identification of Photodegradation Products

**Figure 8:**
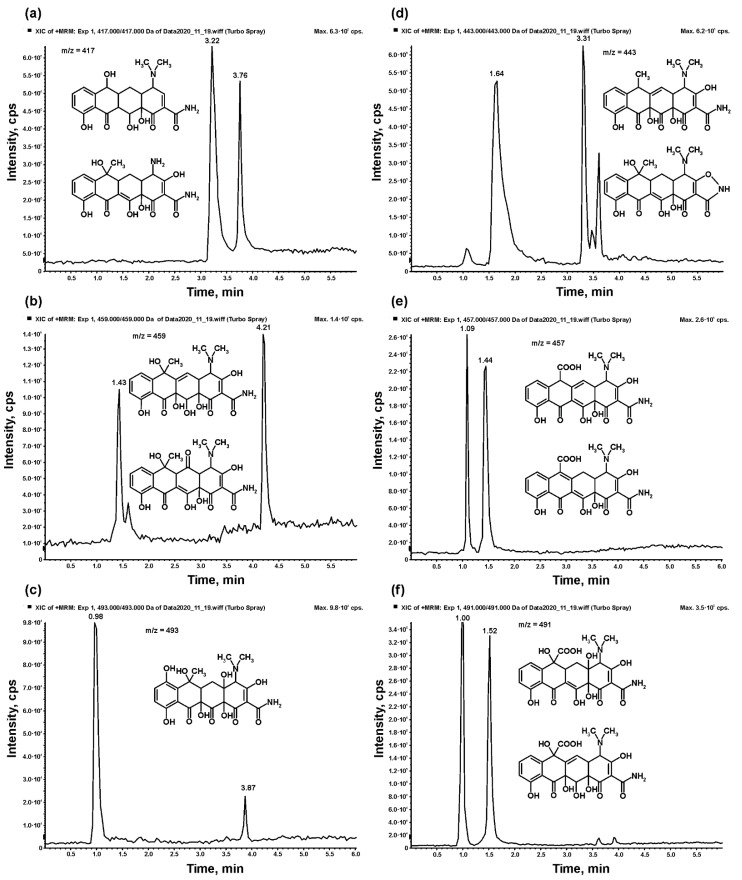
Chromatograms of selected ions and structures of detected tetracycline degradation products at (**a**) *m/z* = 417, (**b**) *m/z* = 459, (**c**) *m/z* = 493, (**d**) *m/z* = 443, (**e**) *m/z* = 457 and (**f**) *m/z* = 491.

The photodegradation products identified in the present study were formed by several different mechanisms. These include the addition or removal of hydroxy groups, removal of methyl groups, dehydrogenation and other modifications of the TC structure (Figure 8). The compounds detected at *m/z* = 417 follow double demethylation in the dimethylamine group, as described by Li and Hu [62], or demethylation in the TC, C-ring with dehydrogenation, as reported by Cao et al. [63]. Both of these compounds at *m/z* = 417 were detected in the tests with TiO_2_, as well as with TiO_2_-ZrO_2_-ZnO.

The addition of a hydroxy group associated with dehydrogenation was also detected during TC degradation in both tests. The ions at *m/z* = 459 were detected, and this is characteristic of the compounds previously described by Li and Hu [62] and Cao et al. [63]. The inclusion of multiple hydroxy groups was confirmed by the presence of ions at *m/z* = 493, which were detected in the test with TiO_2_, as well as with TiO_2_-ZrO_2_-ZnO. Other changes in the hydroxy group positions in the TC molecule were also found in both tests, as ions were detected at *m/z* = 443 and the structure reported by Cao et al. [63] was found. Interestingly, the same ion at *m/z* = 443 was found with a greater retention time in the test with TiO_2_, but its fragmentation showed a considerably different structure. For this compound, no elimination of NH_3_ was noted during the mass spectrometric fragmentation, showing the lack of amide groups in the structure. On the other hand, the fragmentation pattern included the elimination of dimethylamine, which was not favored in the presence of the amide group, as found in all other compounds analyzed in the present study. This compound has not been reported previously.

Moreover, compounds formed after the oxidation of methyl groups to carboxylic groups were also detected. The ions found at *m/z* = 457 and *m/z* = 491 have a characteristic fragmentation pattern, which includes the loss of 44 Da, i.e., the removal of a CO_2_ molecule characteristic of the presence of the carboxylic group. Once again, such compounds have not previously been reported in the degradation of TC.

## 4. Conclusions

The present study has demonstrated that the sol–gel method, assisted by a calcination process, is effective in the fabrication of active TiO_2_-based photocatalysts with tailored physicochemical properties (such as crystalline structure, surface area, pore structures and optical properties). The mentioned properties are strongly dependent on the amounts of components in the product, as well as on the calcination temperature.

The results of the XRD analysis show that the introduction of ZrO_2_ or ZnO into the structure of the TiO_2_-based mixed oxide materials enabled the formation of the photoactive, orthorhombic TiZrO_4_ phase and ZnTiO_3_ phases, which can play a crucial role in the photocatalytic activity of the heterogeneous photocatalysts. 

It was found that the synthesis of mixed oxide systems containing ZrO_2_ in their structure leads to photocatalysts with a higher BET surface area. 

The results of the tests of the photodecomposition of tetracycline in the presence of the fabricated mixed oxide materials under UV–Vis irradiation indicated that the crystalline structure of the heterogeneous photocatalysts, as well as the pH of the reaction system, has a significant influence on the degradation efficiency. In all cases, ternary oxide systems (samples T8Zr1Zn1_600 and T8Zr1Zn1_800) exhibited the highest photocatalytic activity. 

## Data Availability

Not applicable.

## Supplementary Materials

The following are available online at https://www.mdpi.com/article/10.3390/ma14185361/s1, Figure S1. Morphology of synthesized photocatalysts: (a) Ti_600, (b) Ti_800, (c) Ti8Zr2_600, (d) Ti8Zr2_800, (e) Ti8Zn2_600, (f) Ti8Zn2_800, (g) Ti8Zr1Zn1_600 and (h) Ti8Zr1Zn1_800. Figure S2. Surface composition of synthesized photocatalysts. Figure S3. FTIR spectra of: (a) Ti_600 and Ti_800, (b) Ti8Zr2_600 and Ti8Zr2_800, (c) Ti8Zn2_600 and Ti8Zn2_800, (d) Ti8Zr1Zn1_600 and Ti8Zr1Zn1_800 oxide photocatalysts.
