# Supplementary material for: Synthesis of Selected Mixed Oxide Materials with Tailored Photocatalytic Activity in the Degradation of Tetracycline
Source: Materials (Basel). 2021 Sep 17;14(18):5361. doi: 10.3390/ma14185361 (PMC8471185; doi:10.3390/ma14185361)
Supplement: Supplementary file 1 [file materials-14-05361-s001.zip › materials-1312578-supplementary.pdf]

materials

Article

Synthesis of selected mixed oxide materials with tailored photocatalytic activity in the degradation of tetracycline

Katarzyna Siwińska-Ciesielczyk [1,*], Angelika Andrzejczak [1], Dominik Paukszta [1], Adam Piasecki [2], Dariusz Moszyński [3], Agnieszka Zgoła-Grześkowiak [4] and Teofil Jesionowski [1]

[1] Poznan University of Technology, Faculty of Chemical Technology, Institute of Chemical Technology and Engineering, Berdychowo 4, PL-60965 Poznan, Poland; andrzejczak194@wp.pl (A.A.), dominik.paukszta@put.poznan.pl (D.P.), teofil.jesionowski@put.poznan.pl (T.J.)
[2] Poznan University of Technology, Faculty of Mechanical Engineering and Management, Institute of Materials Science and Engineering, Jana Pawla II 24, PL-60965 Poznan, Poland; adam.piasecki@put.poznan.pl
[3] West Pomeranian University of Technology, Szczecin, Faculty of Chemical Technology and Engineering, Institute of Inorganic Chemical Technology and Environment Engineering, Piastów 42, PL-71065 Szczecin, Poland; dmoszynski@zut.edu.pl
[4] Poznan University of Technology, Faculty of Chemical Technology, Institute of Chemistry and Electrochemistry, Berdychowo 4, PL-60965 Poznan, Poland; agnieszka.zgola-grzeskowiak@put.poznan.pl
* Correspondence: katarzyna.siwinska-ciesielczyk@put.poznan.pl; Tel.: +48 61 665 36 26

Citation: Siwińska-Ciesielczyk, K.; Andrzejczak, A.; Paukszta, D.; Piasecki, A.; Moszyński, D.; Zgoła-Grześkowiak, A.; Jesionowski, T. Synthesis of Selected Mixed Oxide Materials with Tailored Photocatalytic Activity in the Degradation of Tetracycline. *Materials* **2021**, *14*, x. https://doi.org/10.3390/xxxxx

Academic Editor: Stefano Lettieri

Received: 8 July 2021
Accepted: 11 September 2021
Published: 17 September 2021

Publisher's Note: MDPI stays neutral with regard to jurisdictional claims in published maps and institutional affiliations.

Copyright: © 2021 by the authors. Licensee MDPI, Basel, Switzerland. This article is an open access article distributed under the terms and conditions of the Creative Commons Attribution (CC BY) license (http://creativecommons.org/licenses/by/4.0/).

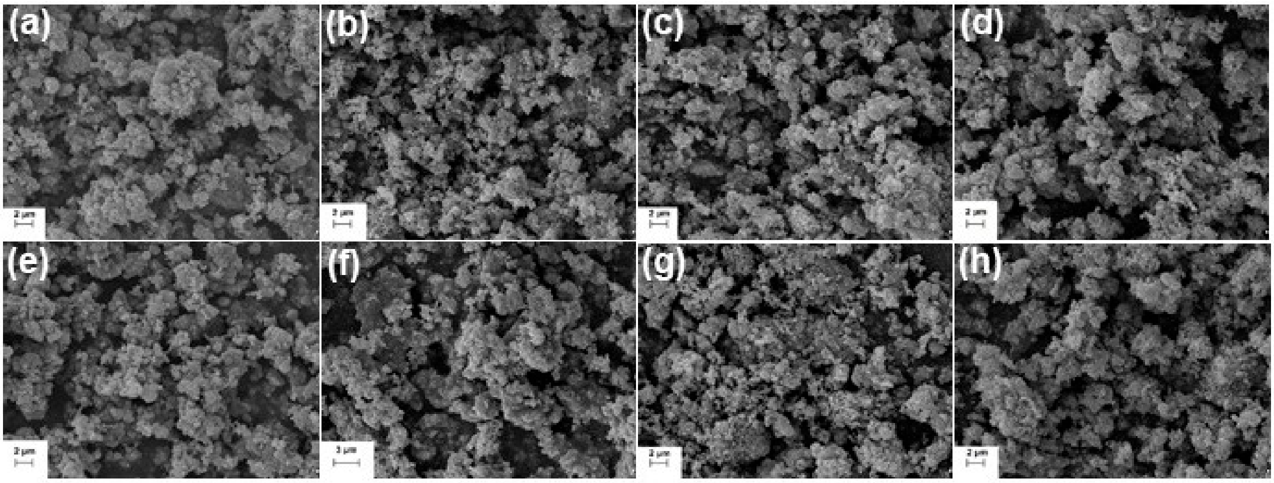

Figure S1. Morphology of synthesized photocatalysts: (**a**) Ti_600, (**b**) Ti_800, (**c**) Ti8Zr2_600, (**d**) Ti8Zr2_800, (**e**) Ti8Zn2_600, (**f**) Ti8Zn2_800, (**g**) Ti8Zr1Zn1_600 and (**h**) Ti8Zr1Zn1_800.

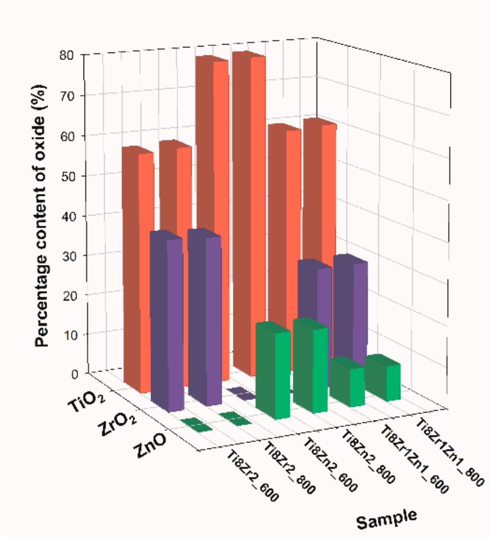

Figure S2. Surface composition of synthesized photocatalysts.

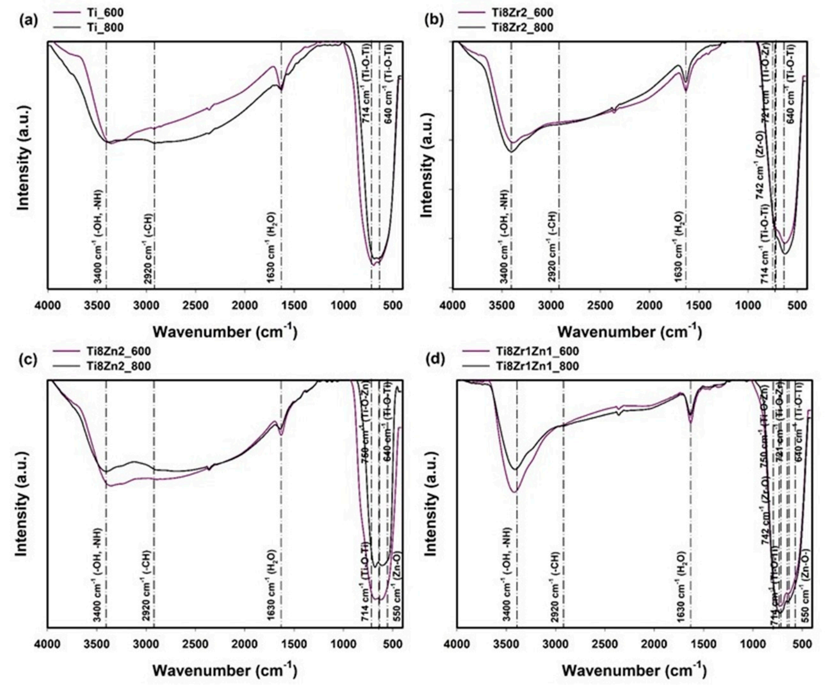

Figure S3. FTIR spectra of: (**a**) Ti_600 and Ti_800, (**b**) Ti8Zr2_600 and Ti8Zr2_800, (**c**) Ti8Zn2_600 and Ti8Zn2_800, (**d**) Ti8Zr1Zn1_600 and Ti8Zr1Zn1_800 oxide photocatalysts.
